# A Comprehensive Machine-Learning-Based Software Pipeline to Classify EEG Signals: A Case Study on PNES vs. Control Subjects

**DOI:** 10.3390/s20041235

**Published:** 2020-02-24

**Authors:** Giuseppe Varone, Sara Gasparini, Edoardo Ferlazzo, Michele Ascoli, Giovanbattista Gaspare Tripodi, Chiara Zucco, Barbara Calabrese, Mario Cannataro, Umberto Aguglia

**Affiliations:** 1 Department of Medical and Surgical Sciences, Magna Graecia University of Catanzaro, 88100 Catanzaro, Italy; giuseppevaronech@gmail.com (G.V.); s.gasparini@unicz.it (S.G.); ferlazzo@unicz.it (E.F.); m.ascoli@neurorc.it (M.A.); chiara.zucco@gmail.com (C.Z.); bcalabresemail@gmail.com (B.C.); cannataro@unicz.it (M.C.); 2 Regional Epilepsy Centre, Great Metropolitan Hospital, 89100 Reggio Calabria, Italy; tripodi.giovanbattistag@gmail.com

**Keywords:** EEG, psychogenic nonepileptic seizures, machine learning

## Abstract

The diagnosis of psychogenic nonepileptic seizures (PNES) by means of electroencephalography (EEG) is not a trivial task during clinical practice for neurologists. No clear PNES electrophysiological biomarker has yet been found, and the only tool available for diagnosis is video EEG monitoring with recording of a typical episode and clinical history of the subject. In this paper, a data-driven machine learning (ML) pipeline for classifying EEG segments (i.e., epochs) of PNES and healthy controls (CNT) is introduced. This software pipeline consists of a semiautomatic signal processing technique and a supervised ML classifier to aid clinical discriminative diagnosis of PNES by means of an EEG time series. In our ML pipeline, statistical features like the mean, standard deviation, kurtosis, and skewness are extracted in a power spectral density (PSD) map split up in five conventional EEG rhythms (delta, theta, alpha, beta, and the whole band, i.e., 1–32 Hz). Then, the feature vector is fed into three different supervised ML algorithms, namely, the support vector machine (SVM), linear discriminant analysis (LDA), and Bayesian network (BN), to perform EEG segment classification tasks for CNT vs. PNES. The performance of the pipeline algorithm was evaluated on a dataset of 20 EEG signals (10 PNES and 10 CNT) that was recorded in eyes-closed resting condition at the Regional Epilepsy Centre, Great Metropolitan Hospital of Reggio Calabria, University of Catanzaro, Italy. The experimental results showed that PNES vs. CNT discrimination tasks performed via the ML algorithm and validated with random split (RS) achieved an average accuracy of 0.97 ± 0.013 (RS-SVM), 0.99 ± 0.02 (RS-LDA), and 0.82 ± 0.109 (RS-BN). Meanwhile, with leave-one-out (LOO) validation, an average accuracy of 0.98 ± 0.0233 (LOO-SVM), 0.98 ± 0.124 (LOO-LDA), and 0.81 ± 0.109 (LOO-BN) was achieved. Our findings showed that BN was outperformed by SVM and LDA. The promising results of the proposed software pipeline suggest that it may be a valuable tool to support existing clinical diagnosis.

## 1. Introduction

Psychogenic nonepileptic seizures (PNES) are sudden behavioral changes mimicking epileptic seizures without ictal electroencephalography (EEG) changes [1,2]. PNES have been linked to dysfunction in the processing of psychological or social distress, abuse during childhood, or severe traumatic events [3,4]. The prevalence of PNES is high in selected populations, such as 5–20% in outpatient epilepsy populations [5,6] and 10–40% in patients referred to tertiary epilepsy centers for medically refractory seizures [1,7,8]. Misdiagnosis of epilepsy may lead to treatments with antiepileptic drugs (AEDs), posing the risk of iatrogenic morbidity and elevated cost for the healthcare system [9]. The gold standard for PNES diagnosis is the visual examination of clinical events [2,3,10] captured during video EEG, either occurring spontaneously or provoked by suggestion techniques. These methods are time-consuming and ethically disputable [11]. Using a consensus review of the literature, the International League Against Epilepsy (ILAE) evaluated key diagnostic approaches, including detailed history and seizure description, EEG recordings, video EEG monitoring, neurophysiology, neuroimaging, hypnosis, and neurohumoral monitoring [9]. It has been found that PNES diagnosis can have a long diagnostic delay [5]. This could be partly due to the fact that EEGs from patients with PNES do not show any abnormal electrophysiological pattern in time-domain EEGs. To overcome this limitation, during the last decade, a plethora of machine learning (ML) techniques and algorithms have been widely used to predict significant discrimination tasks of the disease. ML algorithms perform good discrimination tasks in small datasets [12]. To augment ML performance, feature extraction techniques are used. Feature extraction removes irrelevant patterns and achieves a reduction of dimensionality and noise, increasing the robustness of the learning model. There have been several EEG classification studies in recent years. These studies have implemented many different classification models, compared their performance, and measured distinct combinations of feature sets. Among these classifiers, linear discriminant analysis (LDA), support vector machine (SVM), and Bayesian network (BN) have been popular [13,14]. In [15], Morabito et al. extracted different statistical features, such as mean (µ), standard deviation (σ), and skewness (v), from nontraditional sub-bands in the time-frequency maps of EEG signals. In many studies [15,16,17,18], the statistical features µ, σ, and v provided very robust classification scores. Using artificial intelligence (AI) algorithms, Gasparini et al. [15] were able to discriminate EEG time series of PNES from healthy controls. Based on previous results [15], in this paper we propose a ML pipeline to classify PNES from healthy controls (CNT) via statistical features extracted from a spectral EEG map. In the light of these previous results, we decided to use the mean, standard deviation, skewness, and kurtosis (k). Our feature vector was fed into the three different supervised ML algorithms, i.e., SVM, LDA, and BN, to perform EEG epoch classifications tasks for PNES vs. CNT.

## 2. Materials and Methods

### 2.1. Experimental Protocol and Dataset Description

The data analyzed in this work were collected from the Regional Epilepsy Centre, Great Metropolitan Hospital of Reggio Calabria, University of Catanzaro, Italy. In this study, we analyzed 10 EEG time-series recordings from 10 patients (2 males, age 28 ± 12.4) with PNES and 10 CNT subjects (3 males; age 33 ± 13.93). Patients were recruited successively among those referred to our center; EEG recordings with too many artifacts were excluded. Healthy controls were also recruited prospectively during the same period among patients’ caregivers or healthcare personnel. PNES diagnosis was made based on a typical episode recorded during video EEG, occurring either spontaneously or in response to suggestion maneuvers, with EEG showing neither concomitant ictal activity nor post-ictal (diagnostic EEG). Healthy controls did not suffer from any neurological disorder and had a normal neurological examination. None of the 20 subjects were on chronic medication or had received any drug in the previous 24 h before EEG acquisition. The study was conducted following the Declaration of Helsinki and formally approved by the local Medical Research Ethics Committee.

### 2.2. EEG Acquisition

EEG recordings utilized for the present study were conducted in a poorly lit room using 19 Ag/AgCl surface electrodes placed according to the International 10/20 system. These EEGs were recorded 10 to 20 min before the diagnostic EEG. Recordings were performed with a Micromed Brain Quick system (Micromed SpA, Mogliano Veneto (TV), Italy) with a sampling rate of 512 Hz, high-pass filter at 0.5 Hz, low-pass filter at 70 Hz, plus a 50 Hz notch filter with a slope of 12 dB/Oct at 512 bit/second. All of the EEG signals were recorded using a montage with the following channel layout: Fp1, Fp2, F3, F4, C3, C4, P3, P4, O1, O2, F7, F8, T3, T4, T5, T6, Fz, Cz, and Pz and reference in G2 (located between electrodes Fz and Cz). All the electrode skin impedance values were kept below 5 KΏ. The EEG data were recorded in a resting condition for 20 min.

Participants were comfortably seated in a reclining chair with their eyes closed. The technicians kept the subjects alert to prevent drowsiness. At the end, EEGs were downsampled to 256 Hz, segmented into 20 min long records, and stored on an optical disc in the American Standard Code for Information Interchange (ASCII) format for further processing. The EEG recordings were later manually reviewed by experts in order to cancel the segments affected by artifacts. 

### 2.3. EEG Software Pipeline

**Figure 1:**
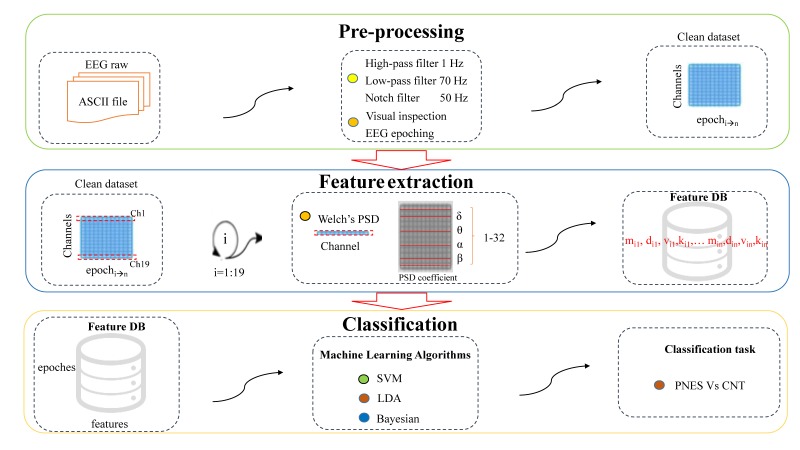
Software pipeline.

In this section, the proposed ML pipeline is briefly described. In Figure 1, the architecture of the proposed approach is pictorially described. Our database included PNES and CNT subjects that were processed in multiple steps as follows.

- Artifact rejection: Artifactual EEGs were rejected through visual inspection. At this stage, to avoid imbalances, the dataset size was changed from 20 to 15 min.
- Signal filtering: EEG was high-pass filtered at 1 Hz and low-pass filtered at 70 Hz plus notch at 50 Hz.
- EEG epoching: The artifact-free epoch EEG recordings were segmented in nonoverlapping T = 5 s epochs.
- Power spectrum analysis: The spectral structure of a sliding window of length L = 5 s was extracted. We used the Welch method and sliding Hamming window with 50% overlap on 1280 samples for the segment.
- Feature extraction: The power spectral density (PSD) of the epochs was split into five submaps corresponding to the five main EEG sub-bands: delta (1–4 Hz), theta (4–8 Hz), alpha (8–13 Hz), beta (13–32 Hz), and the whole band (1–32 Hz). After that, given the PSD submap of the EEG band under analysis, four features were extracted: mean (m), standard deviation (d), skewness (v), and kurtosis (k). Hence, 5 (sub-bands) × 4 (features) = 20 PSD features were extracted for each EEG epoch.
- Dataset preparation: A stacked feature vector XT was the result of concatenating contiguous W feature vectors in nonoverlapping PSD sliding windows. Therefore µ, σ, v, and k extracted from four frequency sub-bands and µ, σ, k, and v of the whole PSD map were concatenated in XT. The feature vector output size for each subject was of 4 (features) × 5 (frequency bands) × 19 (electrodes) × 120 (epochs) = 45,600 elements.
- Classification algorithm: The framework implemented three different ML approaches, namely, SVM, BN, and LDA, in order to discriminate EEG time series of PNES patients from the CNT ones.

### 2.3.1. EEG Preprocessing

All EEG data were manually reviewed by epileptologists to label and reject the artifactual time series. Two epileptologists independently reviewed all the data to remove artifacts such as (i) eye blinking, (ii) muscular movement, (iii) heart rate, and (iv) sensor artifact. Afterwards, the EEGs were bandpass-filtered between 1 and 70 Hz with the 3rd order of Butterworth bandpass filter plus notch (50 Hz) in order to include delta (1–4 Hz), theta (4–8 Hz), alpha (8–13 Hz), beta (13–32 Hz), and the whole band (1–32 Hz), which were the most important EEG rhythms. At the end of the 20 min, artifact-free time series were finally selected for each subject and segmented into nonoverlapping EEG epochs sized L = 5 s. Each EEG epoch was of L = 5 × 256 = 1280 samples. The EEG epochs were preprocessed one by one with handwritten Matlab 2018a (The MathWorks, Inc., Natick, MA, USA) algorithms, then stored as .mat files for further analysis.

### 2.3.2. EEG Feature Extraction

Our EEG database, described in Section 2.1, included 10 CNT subjects and 10 PNES patients. In this study, the classification was based on the four statistical features that were extracted from individual artifact-free time series recorded during EEG periods in resting state and with eyes closed. All features were computed in 5 s nonoverlapping epochs selected from artifact-free EEG segments. From each EEG dataset, 120 nonoverlapping time windows (epochs) of 5 s were extracted. Using this approach, our dataset held (19 × 120 × 4 × 5) features for each subject. The total dataset size was [10 × (19 × 120 × 20) + 10 × (19 × 120 × 20)] = 9600 × 304 features. For each EEG epoch (channels × epoch length, 19 × 1280), the PSD was evaluated for each channel. Each PSD map was partitioned into five submaps corresponding to the five nonoverlapping sub-bands that were analyzed (delta (1–4 Hz), theta (4–8 Hz), alpha (8–13 Hz), beta (13–32 Hz), and the whole band (1–32 Hz)) frequencies. In each submap, the mean (m), standard deviation (d), skewness (v), and kurtosis (k) were estimated. The selected features have been successfully validated in [16,17,18]. The employed features have been extensively described in previous sections. At the last vector size is 5 (# sub-bands) × 4 (# features) = 20 features. The 5xn value was stacked into 1 × n feature vector X_T_, with n = 5 × 120. At the end, for each subject, we had a X_T_ size equal to channel × epoch × feature. This consisted of 19 × 120 × 4 × 5 = 45,600 features for each subject under analysis. The power spectral density is defined as the Fourier transform (FT) of the signal’s autocorrelation function. In this paper, the Welch method was applied along with a Hamming window [19]. The Welch approach splits the times series into overlapping chunks, computing a modified periodogram of each chunk, and the PSD estimates are then averaged. The PSD estimates were obtained in delta, theta, alpha, beta, and the whole frequency band for each EEG sensor. Furthermore, the µ, σ, k, and v from the spectral map was obtained for each band. Due to the entry EEG sequence x_i_(n), we chose the follow representation:

x_i_ (n) = x(n + ih), n = 0, 1, 2, 3, …..M-1

with i = 0,1,2,3,…..L-1

where n is the number of windows, h is the window’s length, and M represent the maximum number of segments. The output of periodogram can be represented as follows:

$$P_{xx}\left(f\right)=\frac{1}{MU}{\left|\overset{M-1}{\underset{n=0}{\sum }}x_{i}\left(n\right)\omega \left(n\right)e^{-j2\pi fn}\right|}^{2}$$

where *U* gives the normalization factor. The Welch power spectrum is as follows:

$$P_{xx}^{W}=\frac{1}{L}\overset{L-1}{\underset{n=0}{\sum }}P_{xx}\left(f\right).$$

The *P_xx_* is intended for continuous spectra. The PSD carries out the average energy embedded in the signals in each frequency band under analysis. Then, integrating the PSD value, the power spectral density can be computed. To do this, we used Matlab function (cit.).

[*P_xx_, f*] = psd(x, nfft, fs, window)

where “x” specifies the input sequence, and “nfft” specifies the length of the fast Fourier transform (FFT) to perform on each EEG segment. The EEG segment length was made up of 256 samples. The length of the “window” must be less than or equal to “nfft”. The Hamming window was set with the same length as “nfft”. The function output returned the power spectrum of the input signal “x”.

### 2.3.3. EEG Feature Classification

Given an ith EEG epoch, in order to classify it as CNT or PNES, three different ML classifiers, namely, SVM, LDA, and BN, were used, the parameters and results of which are presented in the next section.

#### Support Vector Machine

SVM [12] is a flexible and powerful statistical learning tool for binary classifier. SVM works by construction of a N-dimensional hyperplane that optimally separates the data into two categories. SVM maps the data into a higher dimensional space and then constructs an optimal separating hyperplane in this space. SVM can deal with large feature spaces. An input classifier row of feature set is called a vector. The most important training stage in SVM is the hyperplane definition.

The aim of classification by the SVM algorithm is to find an optimal hyperplane that separates clusters of vectors into two different nonoverlapping classes. Training an SVM is relatively easy [20]. It works relatively well for high-dimensional feature sets. Complexity and error trade-off can be controlled manually. The choice of a kernel is most important in the trade-off between computational performance and speed of execution. In this way, a SVM kernel function maps the data into a different nonlinear region by a more complex hyperplane. The kernel separates two input vectors by projecting it into higher dimensional space. Later, the support vector method was extended for solving function estimation problems. Given a training set of N data points ${\left\{y_{k},x_{k}\right\}}_{k=1}^{N}$, where $x_{k}\in {\mathbb{R}}^{n}$ is the k^th^ input pattern and $y_{k}\in {\mathbb{R}}^{n}$ is the k^th^ output pattern, the SVM classifier model can be expressed as follows:

$$y\left(x\right)=sign\left[\overset{N}{\underset{k=1}{\sum }}a_{k}y_{k}\psi \left(x,x_{k}\right)+b\right]$$

where $a_{k}$ and b are positive real constants. 

In this study, we used a commonly adopted kernel called the radial basis function (RBF). The scalar product of the two data points x and y under the feature map implied by the RBF kernel is computed as follows:

$$\psi \left(x,x_{k}\right)=exp\left\{-\|x-x_{k}{\|}_{2}^{2}/\sigma^{2}\right\}\;\mathrm{for}\;\mathrm{RBF}$$

where σ is a free parameter.

Once a kernel function is selected, the SVM algorithm works by identifying a hyperplane in a feature space that optimally separates the two classes in the training data, giving the maximum margin between the images in feature space of the points in the two classes. Often it is desirable to allow a few misclassifications in order to achieve a wider margin of separation; this trade-off is controlled by another parameter called the training error cost, which is usually denoted by C.

#### Bayesian Networks

A Bayesian network is a network with directly linked node and probability, random variable, and finite number of state functions attached to each one. The edges represent the relationships between the nodes. The node with no link will contain a small probability of having a parent node. 

We can represent the learning problem as follows: $X=\left\{X_{1}\ldots \ldots \ldots X_{n}\right\}$, where each node *X_i=1_* corresponds to S (direct acyclic graph of a *X* conditional variable set) at the probability set Pa_i_ $\subseteq \left\{X_{1}\ldots \ldots \ldots X_{i-1}\right\}$ corresponding to $X_{1}$ in $S^{1}$. If $S^{h}$ is a hypothesis of structure and $\theta_{s}$ are the hypothesized parameters, then for given a set $D=\left\{x_{1}\ldots \ldots \ldots x_{n}\right\}$ with a random sample $p(X|\theta_{s},S^{h})$, $\theta_{s}$ and $S^{h}$ are the true parameters and structure hypothesis, respectively. We can compute the probability as follows:

$$p\left(S^{h}|D\right)=\mathrm{cp}\left(S^{h}\right)p\left(D|S^{h}\right)=cp\left(S^{h}\right)\int p\left(D|\theta_{s},S^{h}\right)p\left(\theta_{s}|S^{h}\right)d\theta_{s}$$

where *c* is a normalization constant. This Bayesian network method has been discussed by some authors [16]. Each node in the graph represents a random variable, whereas the edges between the nodes represent probabilistic dependencies among the corresponding random variables. The graph dependencies are estimated using known statistical and computational methods. Hence, BNs provide a simple definition of independence between any two distinct nodes. BN is a directed acyclic graph (DAG). The DAG structure is defined by two sets: the set of nodes (vertices) and the set of directed edges. The nodes represent random variables and are drawn as circles labelled by the variable names.

#### Linear Discrimination Analysis

LDA is a well-known algorithm for feature extraction and dimension reduction. It has been widely used in many applications, such as face recognition, image retrieval, microarray data classification, etc. LDA tries to provide more class separability and draws a decision region between the given classes. The LDA classifier is a dimension reduction method, which finds an optimal linear transformation that maximizes the class separability. LDA creates a linear combination of data sets, which yields the largest mean differences between the desired classes. It performs well when the feature vector is multivariate normally distributed in each class group, and different groups have a common covariance.

### 2.4. Use of Classifiers for the Discrimination between CNT and PNES

In the proposed classification framework, we compared PNES patients with healthy controls. The following classification algorithms were applied and compared: (i) LDA, (ii) SVM, and (iii) BN. 

The basic idea of the SVM is to construct a hyperplane that has the maximum margin between CNT and PNES samples. 

For each of these classification methods, an estimator for the misclassification error, such as accuracy, sensitivity, specificity, and receiver operating characteristic (ROC), was computed through two methods: (i) random split (RS) and (ii) leave-one-out (LOO) cross-validation. LOO cross-validation is often used to estimate the generalization ability of the classifiers. We used leave-one-out cross-validation to achieve greater classifier robustness and to improve the classifier’s ability to generalize new data [15]. In multiple data splits, the likelihood of erroneous results was reduced. Let us define M as the number of chunk and N as the number of epochs, where (N_i=1_ = 120 epoch = 1 subject). Cross-validation was performed by dividing the data into M = 17 splits.

This approach repeatedly trains the classifier on the $N_{i=M-1}$ point and then tests the remaining last one. In the random split approach, our database was split between a training and a test set where training and test sets are respectively 70% and 30%, respectively. A training set was designed as stacked PNES and CNT epochs. We used M = 7, which was randomly extracted from our database. M × N chunks were randomly extracted from the PNES and CNT cohorts. At the end, we designed a training set of 2 (number of class) × 7 M × N = 1,680 epochs. Then, we trained $M_{i=7}$ through separate SVM, LDA, and BN models. The remaining trial was then used as a blind test. The test classification outcome results then came from the accuracy of classifying the respective unseen 6 × N, and the score was averaged across the $N_{i=1:6}$ splits. 

**Figure 2:**
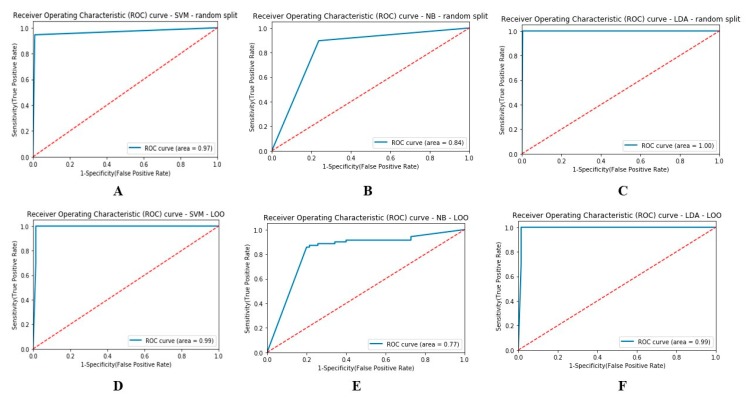
Receiver operating characteristic curve (ROC) of three classifiers, namely, support vector machine (SVM), Bayesian network (BN), and linear discriminant analysis (LDA), (simulated data) with different area under the curve (AUC) values: (**A**,**D**) SVM, (**B**,**E**) NB, and (**C**,**F**) LDA. The classifiers were evaluated through two different methods: (**A**–**C**) random split and (**D**–**F**) leave-one-out (LOO).

All classification outcomes are reported here as the average of all M cross-validation splits. Classification performance can be achieved as a percentage of correctly classified class. A ROC plot [21] is commonly used as a summary for assessing the trade-off between sensitivity and specificity, and the area under the curve (AUC) is used to depict sensitivity and specificity as an indicator for the quality of separation. In this study, for each step, the classification outcome was compared to a threshold, and the subject’s epoch was classified as CNT or PNES as a function of threshold. Using simulated data, Figure 2 shows a comparison of the ROC curve and AUC value obtained from the three different classifiers for PNES and CNT. The ROC of a perfect classifier will go from the bottom left corner via the top left to the top right corner.

LDA, SVM, and BN require many observations as variables. They implement a shallow representation with low computational cost. SVM is the most powerful classification method [22] but prone to overfitting. For the SVM classifier, the following parameters were chosen (kernel = radial, degree = 3, gamma = 0.01, coef = 0, epsilon = 0.1). In contrast, we used the default parameters for LDA and BN. All the classification stages were implemented in Python 3.7 with support from the sklearn library. 

The LDA classifier generated probability discrimination values as PNES patients or CNT. To test the proposed ML approach, we used AUC, sensitivity, specificity, and accuracy. We followed a leave-one-out cross-validation approach for each subject. We used random split validation for the feature database to randomly split between a training and a test set with the proportions of 70% and 30%, respectively. The training set was used to determine the classification model, and the test set was used to evaluate it. 

## 3. Results

In this study, a dataset of 20 EEG signals (10 PNES and 10 CNT) was used. We classified the EEGs as either CNT or PNES based on three different ML models. Given the ith EEG time series, it was first preprocessed, split into 5 s nonoverlapping EEG epochs, and then PSD-transformed into PDS map (where s depended on the EEG length, i.e., 1280 samples). Then, given one PSD map under analysis, 20 statistical features were extracted (as described in Section 2). Afterwards, we designed the whole dataset as the number of epochs × number of features = 9600 × 304, with each of the 120 epoch groups corresponding to one subject. 

For both datasets (CNT and PNES), the classification performance for each classifier was defined as the percentage of correct and incorrect classified patients and healthy controls, considering sensitivity, specificity, F1, and recall, as summarized in Table 1.

Our results demonstrated that all the classification algorithms performed nearly equally well with a remarkable sensitivity of up to 80% in task discrimination among CNT and PNES. In CNT vs. PNES class separation, the NB with LOO, as show in Table 1, was outperformed by LDA and SVM. Indeed, the AUC in Figure 2E is a little bit low compared to the ROC curve obtained by means of a random split (see Figure 2B). The ROC curve was formed by averaging the model’s outcome for each iterative step. However, the low error rate in misclassifications is noteworthy. 

In this study, a binary epoch CNT vs. PNES separation by means of ML algorithms was implemented. The classification performances were evaluated, such as accuracy (ACC), F1 score, recall (RC), and precision (PR) metrics, which are defined as follows:

$$\mathrm{Accuracy}=\frac{\left(\mathrm{TP}+\mathrm{TN}\right)}{\left(\mathrm{TP}+\mathrm{FP}+\mathrm{TN}+\mathrm{FN}\right)};$$

$$\mathrm{Precision}=\frac{Tp}{\left(Tp+Fp\right)};$$

$$Recall=\frac{TP}{Tp/\left(Tp+Fn\right)};$$

$$F1=\frac{2*\left(Precision*recall\right)}{\left(precision+recall\right)};$$

where true positives (TP) and true negatives (TN) indicate test samples correctly classified as PNES or CNT subjects, whereas false positives (FP) and false negatives (FN) indicate the number of test examples that were wrongly detected as subjects with disease and no disease, respectively. Precision is a measure to quantify the number of correct identifications of PNES patients. Recall is a measure of the ability of the classifier to find all the CNT samples. Accuracy is the average of the sensitivity and specificity of the classification. 

We compared the performance of the previously described classifiers through the use of two validation methods, i.e., LOO and random split validation. Table 1 report the evaluation score of the classifier performance. Indeed, SVM reported quite similar scores for the two validation methods. As can be seen in Table 1, the SVM with random split achieved a PR of 0.95 ± 0.03, RC of 0.99 ± 0.001, and ACC of 0.97 ± 0.013. Meanwhile, as can be seen in Table 1, the LOO SVM showed a PR of 0.98 ± 0.061, RC of 0.98 ± 0.126, and ACC of 0.98 ± 0.0233. We statistically evaluated the significant differences in class discrimination among the three classifiers, and the results are reported here as mean ± standard deviation. The SVM classifier showed a likelihood ratio in accuracy for LOO and random split validation equal to 0.975 ± 0.018. Similar evaluation was achieved for LDA with 0.99 ± 0.008 and BN with 0.83 ± 0.05. Furthermore, in order to assess and confirm the ability to correctly detect the EEG epochs of PNES and CNT, the AUC was also estimated. Specifically, Figure 2A–F reports the ROC curves and AUC values of each discrimination technique. The best performance was observed with the LDA classifier (AUC score of 0.990 ± 0.002), as shown in Figure 2C. 

## 4. Discussion and Conclusion

In this work, we propose a novel ML pipeline to automatically classify the rest EEG data of PNES patients and healthy subjects. We carried out many tests in order to determine whether the proposed machine learning tool is robust and flexible. In EEG classification, SVM, LDA, and BN have previously been shown to have good performance in many contexts [14]. For this reason, we sought to determine whether EEG data in our experiment could be automatically classified using the proposed ML pipeline. We exploited the potential of the three different machine learning algorithms to differentiate EEG segments (i.e., epochs) of CNT and PNES. Data-driven ML framework based on PSD representation of EEG recording was proposed. Here, the PSD was used on segmented EEGs (1280 sample in each segment), and features such as the mean (m), standard deviation (d), skewness (v), and kurtosis (k) were extracted from five nonoverlapping EEG rhythms. The extracted feature vector was input as the proposed shallow ML algorithm (SVM, LDA, and BN) to perform binary EEG epoch classification of CNT vs. PNES. Experimental results showed that the LDA achieved better classification performance compared to SVM and BN. This was possible due to the fact that feature extraction was used to reduce redundancy of high-dimensional input data. Here, an input vector of only 380 features for each EEG partition was used to feed the ML classifier. However, it has to be noted that it is difficult to compare our results with other outcomes due to the paucity of other recent studies on EEG epoch-based classification of PNES data. The proposed feature extraction method and the developed ML classifier achieved very good discrimination accuracy score. Our outcomes are quite similar to the result shown in [15], in which Gasparini et al. used a deep learning model to achieve around 90% sensitivity and specificity in PNES discrimination. However, their model is computationally more complex and time-consuming, while their stacked multilayer architecture needs larger datasets to be validated to avoid overfitting and reduced performance. If the ultimate goal is to transfer an algorithm for early and effective diagnosis of PNES to chips, then shallow ML architectures can be used. They avoid bottlenecks in the calculations and reduce cost and calculation time, guaranteeing lower energy expenditure for diagnostic devices to support standalone clinical activity. Nevertheless, the proposed pipeline has some limitations. One of the main limitations is related to the limited patient cohorts. This causes a constrain in classification training/testing performance. A second limitation may be the limited number of features used. A possible improvement could be to increase the dataset dimension and the number of features, for example, by extracting many other features, such as magnitude squared cross power spectral density and mutual information between pairs of sensors, in all the frequencies under analysis. Moreover, this is a cross-sectional study as each subject underwent a single EEG. Longitudinal studies could be performed to test the robustness, stability, and performance of our ML pipeline.

## Figures and Tables

**Table 1:** The classification performance of psychogenic nonepileptic seizures (PNES) vs. health controls (CNT) in terms of precision (PR), recall (RC), and F1 score. We tested the discrimination performance among class labeled as CNT and PNES. The results obtained are quite comparable between the validation methods. The classifier performance provides us with values for each class under analysis.

**Class**	**SVM Classifier (LOO)**		
	Precision	Recall	F1 score
CNT	1.00	0.98	0.99
PNES	0.98	1.00	0.99
**Class**	**LDA Classifier (LOO)**		
	Precision	Recall	F1 score
CNT	1.00	0.98	0.99
PNES	0.98	1.00	0.99
**Class**	**NB Classifier (LOO)**		
	Precision	Recall	F1 score
CNT	0.87	0.75	0.81
PNES	0.78	0.89	0.83
**Class**	**SVM Classifier (Random Split)**		
	Precision	Recall	F1 score
CNT	0.95	0.99	0.97
PNES	0.99	0.95	0.97
**Class**	**LDA Classifier (Random Split)**		
	Precision	Recall	F1 score
CNT	1.00	1.00	1.00
PNES	1.00	1.00	1.00
**Class**	**NB Classifier (Random Split)**		
	Precision	Recall	F1 score
CNT	0.89	0.76	0.82
PNES	0.78	0.90	0.83
**Classifier**	**Random Split Evaluation**		
	Accuracy (ACC)	Precision (PR)	Recall (RC)
SVM	0.97 ± 0.013	0.95 ± 0.03	0.99 ± 0.001
LDA	0.99 ± 0.02	0.99 ± 0.30	0.99 ± 0.053
NB	0.82 ± 0.109	0.83 ± 0.027	0.87 ± 0.163
**Classifier**	**LOO Evaluation**		
	Accuracy	Precision	Recall
SVM	0.98 ± 0.0233	0.98 ± 0.061	0.98 ± 0.126
LDA	0.98 ± 0.124	0.98 ± 0.012	0.98 ± 0.002
NB	0.81 ± 0.109	0.81 ± 0.032	0.81 ± 0.142
